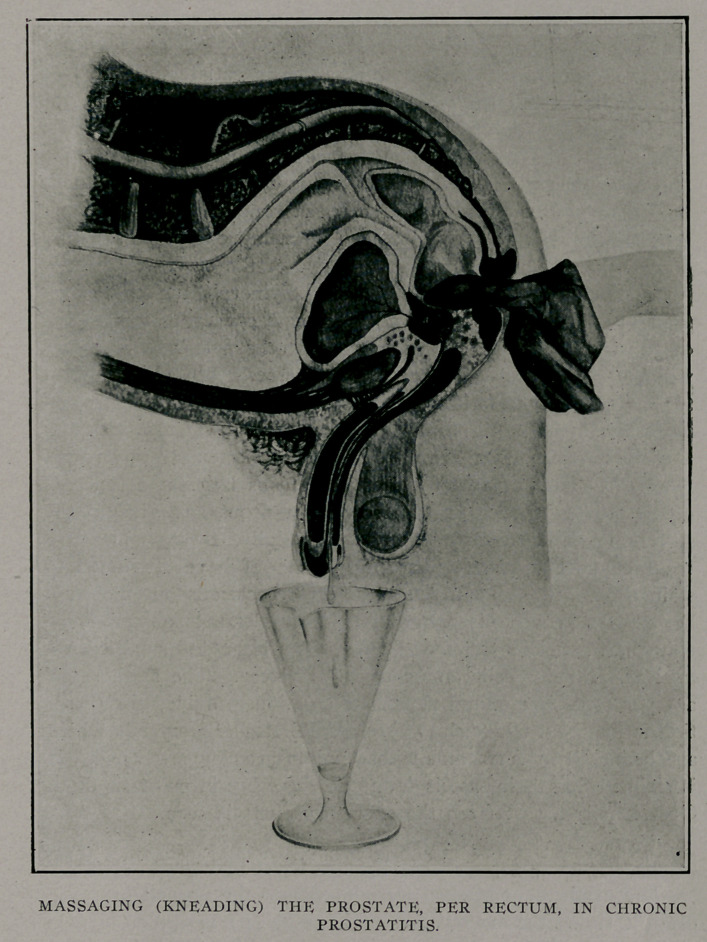# Treatment of Chronic Prostatitis

**Published:** 1910-06

**Authors:** Alfred L. Fowler

**Affiliations:** Atlanta, Ga.; Professor of Genito-Urinary Surgery in the Atlanta College of Physicians and Surgeons; Genito-Urinary Surgeon to Grady (City) Hospital; Surgeon to St. Joseph’s Infirmary; Physician and Surgeon United States Penitentiary Hospital; Member American Urological Association, Etc.


### TREATMENT OF CHRONIC PROSTATITIS.*

BY ALFRED L. FOWLER, M. D., ATLANTA, GA.

*Professor of Genito-Urinary Surgery in the Atlanta College of Physicians and Surgeons; Genito-Urinary Surgeon to Grady (City) Hospital; Surgeon to St. Joseph's Infirmary; Physician and Surgeon United States Penitentiary Hospital; Member American Urological Association, Etc.*

Chronic prostatitis! The most frequent, the most persistent, and also the least frequently recognized of all the complications attending gonorrhœa.

Our earliest description of this affection dates back to the year 1815, when Lognean first described chronic inflammation of the prostate as a gonorrhœal complication. And many years elapsed before any special attention was directed to the disease.

In a treatise on involuntary seminal losses, twenty-one years later, Lallemand pointed out the improvement observed in these cases when treated by posterior instillations and hygiene. He was the first to comprehend the connection between mental and nervous diseases as associated with lesions of the prostate and seminal vesicles. Deslandes, a year later, and his observations were shortly verified by many others, became convinced that *coitus reservatus*, masturbation, ungratified sexual desire and other venereal abuses were etiological factors in chronic prostatitis.

No accurate knowledge of the pathology of the prostate was known until 1838, when Verdier conducted, upon a patient who had long suffered from symptoms of chronic prostatitis, the first autopsy pertaining to the disease. Up to this time the lesions of the prostate and seminal vesicles were not only confounded but they were pretty generally regarded as identical. The findings that were disclosed as a result of this autopsy served to partially explain the confusion.

Careful clinical observations and subsequent researches in this particular branch have cleared up all the apparent contradictions and the independence of the two diseases, vesiculitis and prostatitis, has been established. The researches instituted by such noted urologists as Albarran and Guyon of Paris, Finger of Vienna, Oberlander of Dresden, and Young of Baltimore, have largely increased our knowledge of this disease, both clinically and pathologically.

That this disease is by far more common than was formerly supposed is now pretty generally admitted and its more frequent recognition is due largely to our more precise methods of diagnosis.

ANATOMY.

Just a word rgarding the anatomy of the prostate. First of all, it is essentially a glandular organ, situated below the bladder and tunnelled by the posterior urethra. Two elements enter into its composition:

1. A fibrous connective tissue framework, and unstriped muscular fibres; and

2. Glands, composed of compound branching tubules lined with secreting epithelia ending in short ducts.

The glandular tissue of each lobe consists of twenty or more distinct tube systems, each tube system being drained by an independent duct. The ducts from the lateral lobes enter the posterior urethra in the groove on either side of the vorumontanum, while the ducts from the middle or prosterior lobe enter between the vorumontanum and bladder. This explains how readily the gonococcus invades the prostate.

VIEW, AS SEEN THOUGH THE MICROSCOPE, OF PROSTATIC SECRETION N CHRONIC PROSTATITIS, SHOWING PROSTATIC EPITHELIA, LECITHIN BODIES, CORPORA AMYLACCA AND LEUCOCYTES.

These narrow ducts or tubes, which have their tiny openings in the floor of the posterior urethra, after a course of about 1 mc, pass outward into the lobules whence they divide into repeatedly branching tubules, and expand into the terminal alveoli. By reason of this complicated anatomical arrangement the least disorder results in imperfect drainage from the numerous follicles, and this is why chronic prostatitis lasts so long, and frequently is so difficult to cure.

The arterial supply is from the small branches of the inferior vesical and the middle hemorrhoidal. The vessels of supply enter the periphery of the gland at various points and break up into small capillary net-works that surround the alveoli. The venous supply is very abundant. The sensory nerves of the prostate, somewhat extensive, are derived from the tenth and eleventh dorsal segments, first, second and third sacral and the five lumbar nerves.

In the vast majority of the cases of chronic prostatitis the gonococcus is the offending organism, as is too well borne out by the astounding reports published during the last quarter century. The figures bespeak the frequency which the disease is regarded to occur as a complication of gonorrhœa. From 40 per cent. as reported by Montagnon to 94 per cent. recently reported by one observer, with the better evidence, to my thinking, in favor of the lower figures.

While it is proper to bear in mind the cause of its greatest frequency, nevertheless we should also remember that the disease is not always due to the gonococcus. I have observed it in men who knew not what venereal diseases were, who had been reared in the wilds of the mountains and arrested there by Government officers for illicit distilling and after conviction brought to the United States Penitentiary whence they came under my medical care at this hospital.

Because the disease occurs independently of gonorrhœa may partly explain the high percentage of cases reported by some observers since some of their cases must have had the disease before contracting gonorrhœa. But even so, my clinical experience tells me that these higher figures are incorrect.

The prolonged congestion concomitent to masturbation, excessive venery, coitus reservatus, bicycle riding, etc., without doubt, lowers the power of resistance of the tissue of the gland, and of the prostatic urethra, too, for that matter so that hematogenous infection is rendered comparatively easy. In addition to the blood stream, we have two contiguous reservoirs, the bladder constantly containing urine and the rectum its feces. The colon baccillus is not infrequently found in the urine while the intestine is its natural habitat.

SYMPTOMOLOGY.

Chronic prostatitis develops so insiduously that symptoms, reflex, in distant parts of the body generally go unrecognized. In the author's experience the most constant symptom has been fatigue, and which sometimes amounts to a dull pain, in the lower lumbar region or in the sacro-lumbar region together with lassitude and occasionally melancholia. Other morbid reflex neuroses, not infrequently symptomatic, are:

| | |
|---|---|
| Nocturnal pollutions | Pain in perinaeum |
| Pruritis Ani | Pain in bladder |
| Premature ejaculations | Pain at end of penis |
| Spasmodic sphincter and | Pain in any part of urethra |
| Spasmodic urethral stricture | Pain in kidney region |
| Tickling at meatus | Pain in groin |
| Priapism | Pain in testicle |
| Imperfect erections | Pain in inner side of thighs |
| Erections absent | Pain in legs and feet |

Among the urinary symptoms may be mentioned:

| | |
|---|---|
| Frequent micturition | Weak stream |
| Difficult and painful micturition | Incontinence of urine |
| Burning on urination | Continence of urine |
| Nocturnal urination | Hesitation in urinating |
| Incomplete urination | Hematuria |

In addition to the foregoing, we frequently observe the bon jour drop and prostatorrhœa either on defacation or urination. Of these the latter is the least frequent.

DIAGNOSIS.

By reason of chronic prostatitis, seminal vesiculitis and chronic posterior urethritis having so many symptoms in common, a correct diagnosis can only be made by:

1. Palpating the prostate, per rectum.

THE VERU-MONTANUM, IN CHRONIC PROSTATITIS, AS SEEN THROUGH A GEORGES LUYS URETHROSCOPE. OBSERVE THE EROSIONS AND STRIKING DIFFERENCE AS COMPARED WITH THE NORMAL VIEW.

2. Microscopical examination of the expressed fluid from the prostate.

Before beginning the examination the three glass test of the patient's urine should be made and the findings noted. If the first and last glasses contain Furbringers hooklets it is pretty reliable evidence that the prostate is inflamed. Their absence, of course, does not mean that the gland is normal.

With the patient in the leap-frog position the examining finger on palpation, if educated, detects the absence or presence of enlargement, the lobes and areas involved, whether the diseased and isolated areas are softened and putty-like or nodular and shot-like. The degree of tenderness is also noted. While a very marked involvement, with much nodular and softened areas may easily be felt by an untrained finger, a great deal of experience is necessary to determine the finer points in the diagnosis. If the disease is limited to the prostate, there being no vesiculitis, and no perivesicular infiltration, the sharp outline of the upper border of the prostate can be distinctly felt.

After a systematic examination, the contents of the gland are expressed by actually kneading the prostate with the examining finger and the expression contents are caught in a sterile glass to be examined later with the microscope. This procedure should always be preceded and followed by a copious urethrevesical irrigation.

The color of the normal prostatic fluid varies from a grayish white or yellowish to a bluish white. Its chief characteristic, microscopically, is that it appears as a perfect solution, while the abnormal secretion appears just the contrary. And for this reason the naked eye pathology speaks much to the practiced eye.

Microscopically, the normal secretion is found to contain many lecithin bodies, which are largely responsible for its color, an occasional granule cell, few columnar epithelial cells and now and then corpora amylacea. Bering states that the latter are apparently of epithelial origin and consist of calcium and phosphoric acid.

The secretion as expressed from a chronically inflamed prostate, to naked eye pathology, discloses a heterogeneous fluid. In color it varies from an opalescent white to a creamy yellow. Occasionally it is watery and flaky like the first milk from a punctured cocoanut. Usually it is thick, slimy and contains

VIEWS (NORMAL) OF THE VERU-MONTANUM AS SEEN THROUGH A GEORGES LUYS URETHRASCOPE.

minute masses suggestive of bran or bread crumbs. It is not often that polymorphonuclear cells are found in the homogenous fluid. As a rule the secretion is alkaline.

MASSAGING (KNEADING) THE PROSTATE, PER RECTUM, IN CHRONIC PROSTATITIS.

Microscopy, after all, is the means we must rely upon most for our diagnosis and it should always be resorted to. An occasional pus-cell is, of course, without much significance. The same is true regarding the epithelia unless they are fairly numerous and in a poor state of preservation.

The unstained secretion gives a fairly satisfactory idea of the condition, but the stained specimen more so. Perhaps the simplest and most satisfactory method of staining is to first centrifugate the secretion about twice in a normal salt solution and after decanting or, better still, pipetting off the supernatant fluid stain with methylin blue or green containing about 36 per cent. acetic acid. This will stain everything except the lecithin bodies, the acid accentuating the nuclei of the other cells.

The cells first sought for are the polymorphonuclear and on their presence, so far as the microscope is concerned, hinges the diagnosis. Where there are many pus cells few lecithin bodies will be found. As improvement progresses the pus cells begin to show a decrease while the lecithin bodies become more and more numerous until, finally, a polymorphonuclear is a rarity and the lecithin bodies are profuse. These latter are tiny round refractile bodies of various sizes, seldom as large as a red corpuscle. They appear as opalescent dew-drops and give to the secretion its pearl gray color. Chemically, these bodies are regarded as glycero-phosphate of neurin. I have attempted to stain them with Soudan III on several occasions but the stain has always proved disappointing. Other elements observed are epithelia in various states of preservation, large and small mononuclear, granular cells and corpora amylacea. The addition of a 1 per cent. ammonium phosphate solution, provided the secretion is unmixed with urine, develops Boettscher's crystals which are beautiful in form and whose points are dagger-shaped. It is claimed that their base is found nowhere but in prostatic fluid. Occasionally a cast from the excretory ducts is seen.

URETHROSCOPY.

The urethroscope, when introduced into the normal prostatic urethra, shows the mucous membrane to be of a vermilion or pale pink color, with smooth or somewhat straited folds. The verumontanum shows itself in the floor as a half ball. Occasionally, the tiny openings of the prostatic ducts entering the groove on either side of the verumontanum can be seen. Those from the right lobe enter the groove on that side, those from the left entering the left groove. Less frequently are seen the ejaculatory ducts which enter just anterior to the verumontanum. The appearance of these openings is very similar to the openings of the crypts of Morgagni, which are distributed along the roof of the anterior urethra. The mouth of the sinus pocularis, through which or around which the ejaculatory ducts make their openings, is large and often open.

In the membranous urethra the verumontanum is continued as a more or less prominently projecting ridge of mucous membrane.

Since chronic prostatitis always means a posterior urethritis we may anticipate the change, as disclosed by the urethroscope, in this condition. The verumontanum instead of being a vermilion or pinkish red appears as a congested, succulent purplish red ball showing granular and denuded areas. The mucous membrane not being intact it bleeds from the slightest trauma. Silver nitrate applied to these erosins turn them white. You have observed the same effect when applying silver nitrate to erosions of the bucal cavity.

The cystoscope will generally show a scarlet splotch in the anterior angle of the trigone, while the bougie a boule will serve to detect the usual spasm of the external vesical sphincter present in these conditions.

### TREATMENT.

The successful treatment of chronic prostatitis, even when conducted by those of experience, is not easy. Of prime importance is the recognition that each case is a law unto itself and it should be handled accordingly. Over and too frequent treatment is to be guarded against and an aid not to be lost sight of is gaining the patient's entire confidence.

If we would succeed with these cases we must recognize that diet, next to massage, plays the most important role. That the latter is the sheet-anchor in the treatment. That the intestinal toxemias produce an irritating and aggravating effect in these cases there can be no question, and I have observed it in too many cases for it to be a co-incidence. So I insist on these patients dieting. They are put on a bread and milk diet and directed to drink vichy. Outdoor exercise, baths and rubs after gymnasium practice, together with cold sprays upon the spine are encouraged and which have a happy effect mentally.

Pin-hole meatuses, urethral contractures, spasmodic or organic, should be relieved and varicocele, elongated or adherent prepuces and abnormalities about the rectum or anus should be corrected before beginning the treatment by massage. A urinary antiseptic, such as urotropin, salol or cystogen, has its place in the treatment but the usual tonics, iron, quinine and strychnine are an abomination and a burden to the patient's stomach.

I have not so far felt called upon to employ sero-vaccino-therapy and for two reasons: First, the writer remembers too well the glowing reports heralded in the medical press by those of sophomoric enthusiasm as new and invaluable remedies and which have long been placed at rest in the materia medica graveyard. Second, the plan I am pointing out has proved quite satisfactory in my own hands as well as in the hands of others. The serum-therapy treatment is only an adjunct, at best, and can never supplant massage and diet any more than the Feliki instrument can replace the operator's finger in massaging the prostate.

For the vaccine treatment to be of any benefit it is necessary to use a preparation prepared from the particular organism causing the infection in each individual case and not infrequently we have found in our cultures mixed infections.

The local treatment consists in massaging the prostate, the use of the psychrophore and the Feliki instrument per rectum, copious urethro-vesical irrigations after each massage and direct applications to the diseased posterior uerthra.

The patient is placed in the "leap-frog" position with knees nearly extended while the operator seats himself to the rear and a little to the left of the patient. With a Morton's finger-cot well lubricated on the index finger the operator grasps the patient's left shoulder with his left hand and then inserts his gloved finger with a slight rotary motion its full length. If the prostate is high up placing the foot on the round of a chair with the elbow pressing against the knee insures the operator even a longer reach. After entering the sphincter we can feel the membranous urethra in the median line and to the front; next in the median line may be felt the isthmus between the so-called lateral lobes, while higher up we feel the well-marked border of the prostate. A shallow recess on either side marks the location of the seminal vesicles and the fundus of the bladder is between them. The lateral lobes are on either side the isthmus.

Painful spots, doughy areas and isolated nodules are sought out and noted. The massage as well as the examination should be done with care and deliberation. One lobe at a time is kneaded, not rubbed, with the pulp of the index finger and subsequently pressed out with the entire finger. The degree of pressure employed will depend on the amount of tenderness found. While the whole gland is massaged we direct our particular attention to the nodules and areas of softening. In beginning the massage the outer border of the prostate is selected and our way is gradually worked towards the isthmus and after the entire gland has been dealt with the urethra is stripped and the secretion is collected for a macroscopical and microscopical examination. A copious urethro-vesical irrigation of oxycyanide of mercury, 1-5000, completes the toilet. Generally in four or five days, if the patient has stood the treatment well, another massage is given. In those patients showing a tendency towards epididymitis longer intervals are required and a well fitting suspensory should be worn. No attempt is made to treat these patients chronologically and the number of treatments received within a given time depends altogether on the patient's tolerance.

During the interval between one massage and the next the psychrophore may be employed to good advantage every other day. The hot current continued for 15 or 20 minutes excites an active hyperaemia that aids the absorption of the infiltration. As soon as we detect beginning softening of the nodules or that the soft areas are clearing the hot and cold currents may be used alternately at the same sitting. This greatly stimulates the muscular fibres of the prostate and is particularly useful when neurasthenia is marked. The cooling sound, through which a current of water at the temperature of tap-water passes, is also of much service in selected cases. As soon as a sense of chilliness is produced the instrument should be withdrawn.

Going hand-in-hand with every case of chronic prostatitis is the ever present posterior urethritis and which must receive intelligent treatment. After the prostate has become apparently normal the urethroscope shows the prostatic urethra still unduly congested and which in some instances is due to lesions of the verumontanum, itself. In such instances the verumontanum is swollen, shows granular areas and is purplish red.

While many of the morbid reflex neuroses occurring in urogenital diseases owe their origin to the verumontanum, we must not overlook the fact that such conditions as pin-hole meatuses, varicocele, elongated or adhered fore-skins, vesical and renal lesions also play important roles.

Where the hyperæmia is mild and the erosions of the verumontanums slight, the application of tincture of ioline usually answers pretty well. If the hyperæmia is marked and the granular areas much in evidence a 10 to 20 per cent. silver nitrate application is indicated and if very severe the galvano-cautery should be applied. Subsequent applications should not be made until the reaction has completely subsided, and this is true no matter what the application. Such applications must be applied through the urethroscope directly to the verumontanum.

In closing, out of over fifty specimens of secretions obtained from the prostate in chronic prostatitis, which I sent to Dr. Paulin for examination, the staphylococcus aureus was present in 12 per cent, staphylococcus albus in 26 per cent., staphylococcus citreus in 8 per cent., and the colon bacillus in 10 per cent.; the staphylococcus aureus and coli in 1 per cent.; the aureus and albus in 4 per cent.; citreus and albus 4 per cent. and albus and coli in 6 per cent.

## Figures and Tables

**Figure f1:**
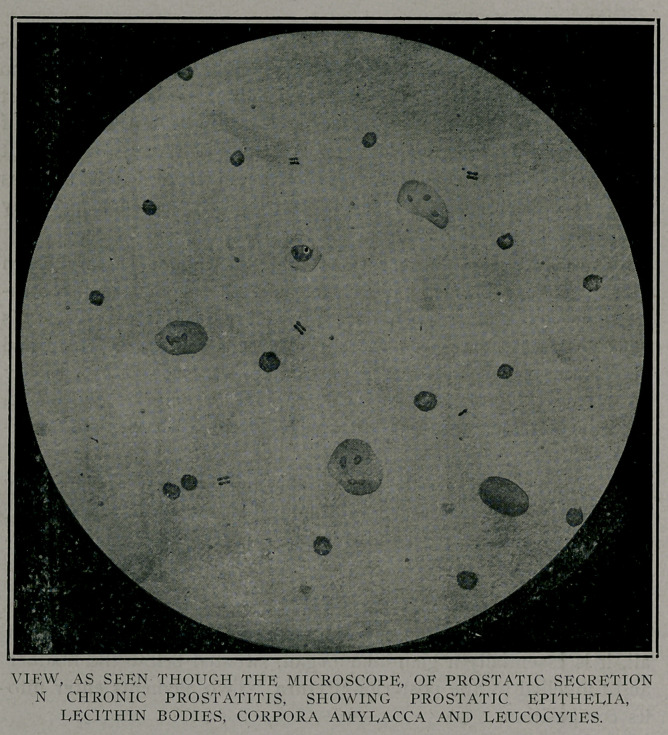


**Figure f2:**
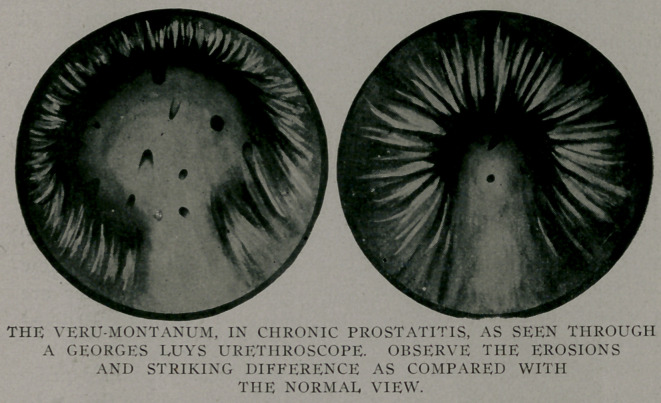


**Figure f3:**
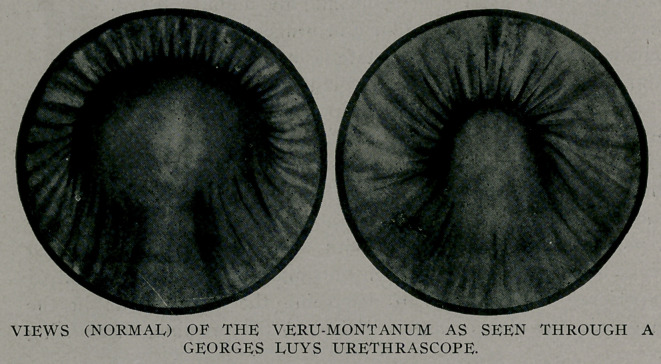


**Figure f4:**